# Simultaneous Detection of the T790M and L858R Mutations in the *EGFR* Gene by Oligoribonucleotide Interference-PCR

**DOI:** 10.3390/ijms20164020

**Published:** 2019-08-17

**Authors:** Keisuke Baba, Toshitsugu Fujita, Sadatomo Tasaka, Hodaka Fujii

**Affiliations:** 1 Department of Respiratory Medicine, Hirosaki University Graduate School of Medicine, 5 Zaifu-cho, Hirosaki, Aomori 036-8562, Japan; 2 Department of Biochemistry and Genome Biology, Hirosaki University Graduate School of Medicine, 5 Zaifu-cho, Hirosaki, Aomori 036-8562, Japan

**Keywords:** EGFR, T790M, L858R, ORNi-PCR, PCR

## Abstract

A de novo single-nucleotide mutation in the *EGFR* gene can cause the development of lung cancer. EGFR tyrosine kinase inhibitors (EGFR-TKIs) are used for clinical treatment of such lung cancers, but acquired resistance often mitigates their efficacy. Accordingly, monitoring of de novo and acquired nucleotide mutations is essential for clinical treatment of lung cancers with EGFR-TKIs. Previously, we reported that oligoribonucleotide interference-PCR (ORNi-PCR) can accurately and cost-effectively detect single-nucleotide mutations. In this study, we applied ORNi-PCR to simultaneous detection of the de novo L858R and acquired T790M mutations in the *EGFR* gene in lung cancer cells. First, we established optimal experimental conditions for ORNi-PCR to simultaneously detect the two single-nucleotide mutations in genomic DNA from lung cancer cells. The conditions we established could also be used for ORNi-PCR using complementary DNA reverse-transcribed from extracted RNA. We found that ORNi-PCR could detect lung cancer cells possessing both single-nucleotide mutations among a large number of cells harboring wild-type sequences, even when the cancer cells constituted less than ~0.2% of all cells. Our findings demonstrate that ORNi-PCR can simultaneously detect multiple single-nucleotide mutations in a gene of interest and might therefore be useful for simultaneous detection of *EGFR* mutations in clinical examinations.

## 1. Introduction

Molecularly targeted therapy has been used for clinical treatment of intractable diseases such as cancers [1]. Tyrosine kinase inhibitors (TKIs) are frequently used in this context [2]. TKIs inhibit the functions of cell-surface receptors that have been mutated into constitutively active forms, thereby arresting oncogenic transformation. Several driver mutations associated with cellular hyper-proliferation have been reported [3]. Mutations in the gene encoding epidermal growth factor receptor (EGFR), a tyrosine kinase receptor belonging to the ErbB family, are well-known drivers of lung cancers. Consistent with this, some EGFR-TKIs dramatically shrink tumors in a subset of patients [4]. EGFR-TKIs suppress proliferative signaling from mutated EGFR [5,6].

Several amino-acid mutations in EGFR have been identified in lung cancers, including Thr790Met (T790M), Leu858Arg (L858R), and partial deletion of the amino acids encoded in exon 19 (Ex19 Del) [7]. L858R and Ex19 Del account for 90% of all *EGFR* mutations [8,9]. T790M is usually observed in acquired resistance caused by selection pressure during clinical treatment with first-generation (gefitinib and erlotinib) and second-generation EGFR-TKIs (afatinib and dacomitinib) [10,11]. In the clinical practice guidelines, the presence of T790M is considered a criterion for prescribing the third-generation EGFR-TKI osimertinib [12,13,14]. Because the types of *EGFR* mutations dictate decisions about treatment with EGFR-TKIs, it is important to identify *EGFR* mutations corresponding to these amino-acid changes in lung cancer cells. Therefore, easy and precise methods for detection of such *EGFR* mutations are essential.

To detect mutations in genomic DNA (gDNA), Sanger sequencing analysis is reliable, but it is time-consuming and its sensitivity is low. Next-generation sequencing (NGS) analysis is a more comprehensive and less biased method for identifying mutations. However, it costs much more and is over-engineered for identification of defined mutations. PCR is the most widely used method for detecting defined mutations in clinical diagnoses [15]. PCR with a specific primer set can distinguish wild-type from mutated sequences. In some cases, however, such primer sets do not work ideally and equally amplify wild-type and mutated sequences. Various PCR-based methods have been developed to avoid ambiguous amplification [16]. For example, blocking PCR using artificial nucleic acids, such as peptide nucleic acids (PNAs) and locked nucleic acids (LNAs) (also known as PNA–LNA clamp PCR), can avoid ambiguous amplification and precisely detect single-nucleotide mutations [17,18]. However, the cost of chemical synthesis of PNAs and/or LNAs is high, potentially increasing the diagnostic cost.

**Figure 1:**
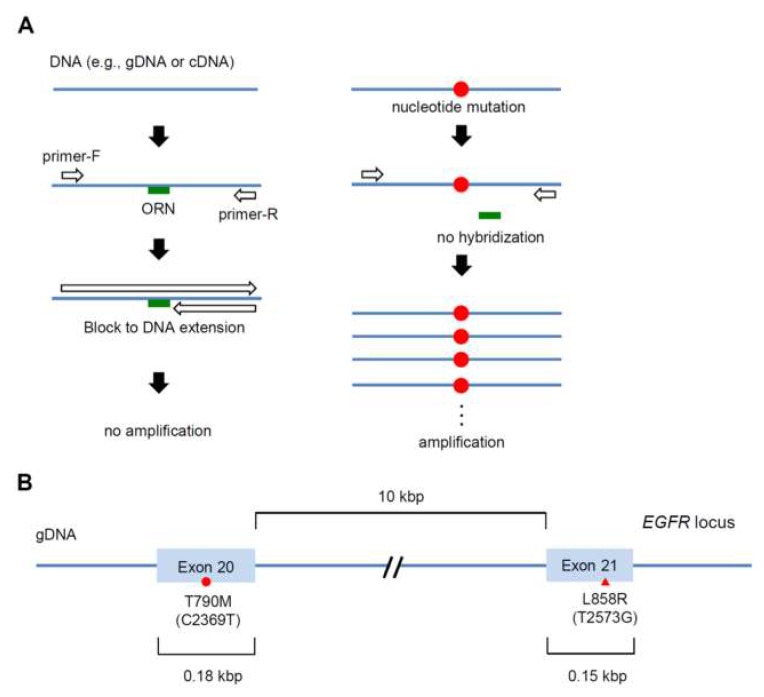
Schematic diagram of ORNi-PCR for detection of single-nucleotide mutations in the human *EGFR* gene. (**A**) Schematic diagram of ORNi-PCR for detection of a single-nucleotide mutation. (**B**) Major clinically relevant single-nucleotide mutations in the human *EGFR* gene. T790M (C2369T) and L858R (T2573G) are in exons 20 and 21, respectively.

We previously demonstrated that oligoribonucleotides (ORNs) can be used to block PCR amplification; we refer to the ORN-based blocking PCR technique as ORN interference-PCR (ORNi-PCR) (Figure 1A) [19]. Chemical synthesis of ORNs is inexpensive, representing an advantage over PNAs or LNAs. In addition, we demonstrated that ORNi-PCR distinguishes single-nucleotide mutations from wild-type sequences with high sensitivity [20,21]. Therefore, ORNi-PCR may be useful for mutation detection in the context of clinical diagnosis. However, it remains unclear whether ORNi-PCR can simultaneously and sensitively distinguish multiple single-nucleotide mutations in one gene in a one-tube reaction.

In this study, we applied ORNi-PCR to simultaneous detection of the two single-nucleotide mutations C2369T (corresponding to T790M) and T2573G (corresponding to L858R) in the same allele of the *EGFR* gene in lung cancer cells (Figure 1B). Our results confirmed that ORNi-PCR could simultaneously detect the two mutations. In addition, we found that complementary DNA (cDNA) was the most suitable template for this purpose.

## 2. Results

### 2.1. Detection of the Single-Nucleotide Mutation L858R (T2573G) by ORNi-PCR

Previously, we demonstrated that ORNi-PCR enables detection of the single-nucleotide mutation L858R (T2573G) in the human *EGFR* gene [21]. We reconfirmed the reported optimal ORNi-PCR conditions with ORN_EGFR_L858, an ORN targeting the wild-type sequence, L858 (T2573). Two-step ORNi-PCR using an annealing/elongation step of 56 or 59 °C completely suppressed DNA amplification of the *EGFR* gene from gDNA extracted from 293T cells harboring the wild-type *EGFR* sequence (Supplementary Figure S1A–D). By contrast, two-step ORNi-PCR amplified the *EGFR* gene from gDNA extracted from NCI-H1975 harboring the L858R (T2573G) mutation in one allele. DNA sequencing analysis confirmed the mutated sequence in the ORNi-PCR product (Supplementary Figure S1E). The melting temperature (T_m_) of ORN_EGFR_L858 was between 59 and 62 °C. These results are consistent with our previous report [21].

### 2.2. Detection of the Single-Nucleotide Mutation T790M (C2369T) by ORNi-PCR

**Figure 2:**
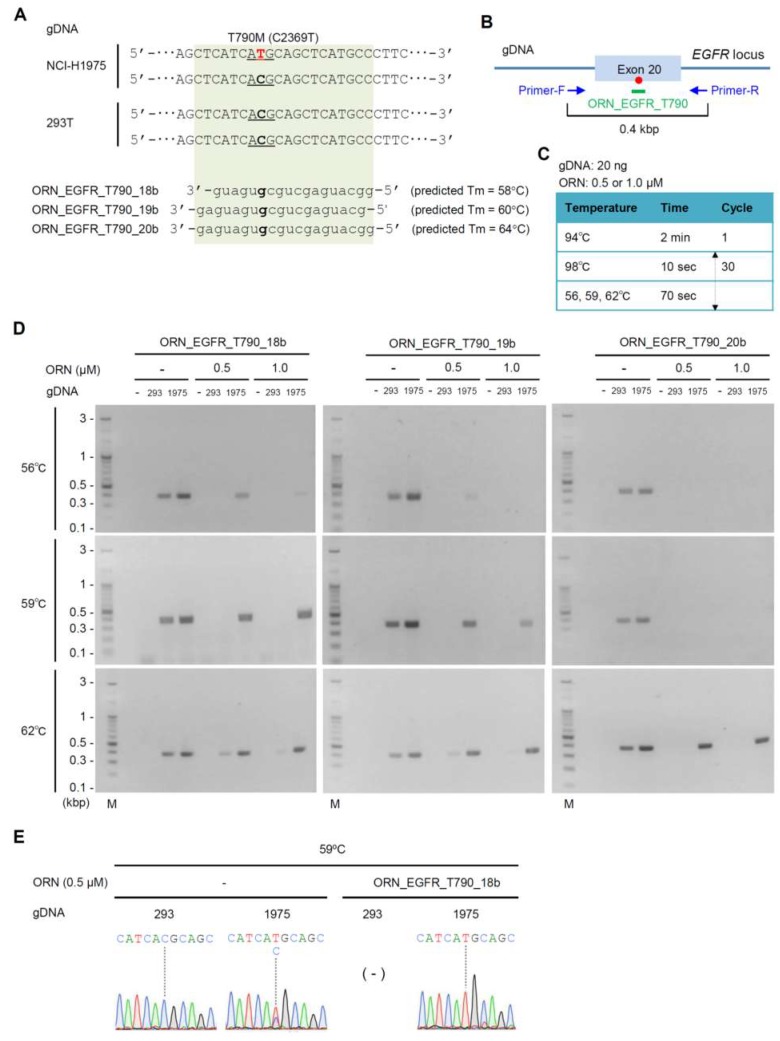
Detection of the T790M (C2369T) mutation by ORNi-PCR. (**A**) ORNs designed for ORNi-PCR. Predicted T_m_ was calculated as reported previously [21]. (**B**) Primer positions for ORNi-PCR. Red circle represents the T790M (C2369T) mutation. (**C**) Conditions for two-step ORNi-PCR. (**D**) Results of ORNi-PCR. (**E**) Results of DNA sequencing analysis. Amplicons of PCR or ORNi-PCR (0.5 µM ORN_EGFR_T790_18b, 59 °C) shown in (**D**) were subjected to DNA sequencing analysis. Sequencing signals around T790 (C2369) are shown.

We next attempted to establish optimal ORNi-PCR conditions for distinguishing the single-nucleotide mutation T790M (C2369T). To this end, we first designed three ORNs targeting the wild-type sequence, T790 (C2369) (ORN_EGFR_T790_18b, ORN_EGFR_T790_19b, and ORN_EGFR_T790_20b; Figure 2A), so that predicted T_m_s would be near the practical T_m_ of ORN_EGFR_L858 (59–62 °C, Supplementary Figure S1D). We then designed a primer set to amplify 0.4 kbp across the ORN target sites (Figure 2B). We used NCI-H1975 gDNA in this experiment because NCI-H1975 harbors the T790M (C2369T) mutation in one allele (Figure 2A). We performed two-step ORNi-PCR using the same reaction conditions as described above for ORN_EGFR_L858 (Figure 2C). As shown in Figure 2D, two-step ORNi-PCR with ORN_EGFR_T790_18b or ORN_EGFR_T790_19b completely suppressed *EGFR* amplification from 293T gDNA, but not NCI-H1975 gDNA, at an annealing/elongation temperature of 59 °C. Two-step ORNi-PCR with ORN_EGFR_T790_20b yielded a similar result at 62 °C. DNA sequencing analysis of the amplicons shown in Figure 2D (0.5 µM ORN_EGFR_T790_18b, 59 °C of the annealing/elongation step) demonstrated that 0.5 µM ORN_EGFR_T790_18b was sufficient to completely suppress amplification of the wild-type *EGFR* sequence (Figure 2E). Thus, we were able to establish optimal conditions for detection of the T790M (C2369T) mutation in gDNA by ORNi-PCR: 0.5 µM ORN_EGFR_T790_18b and annealing/elongation at 59 °C. Considering the practical T_m_ of ORN_EGFR_L858 (59 °C), ORN_EGFR_T790_20b would be unsuitable for combined use with ORN_EGFR_L858 in ORNi-PCR.

### 2.3. Simultaneous Detection of the T790M (C2369T) and L858R (T2573G) Mutations by Multiplex ORNi-PCR with gDNA

**Figure 3:**
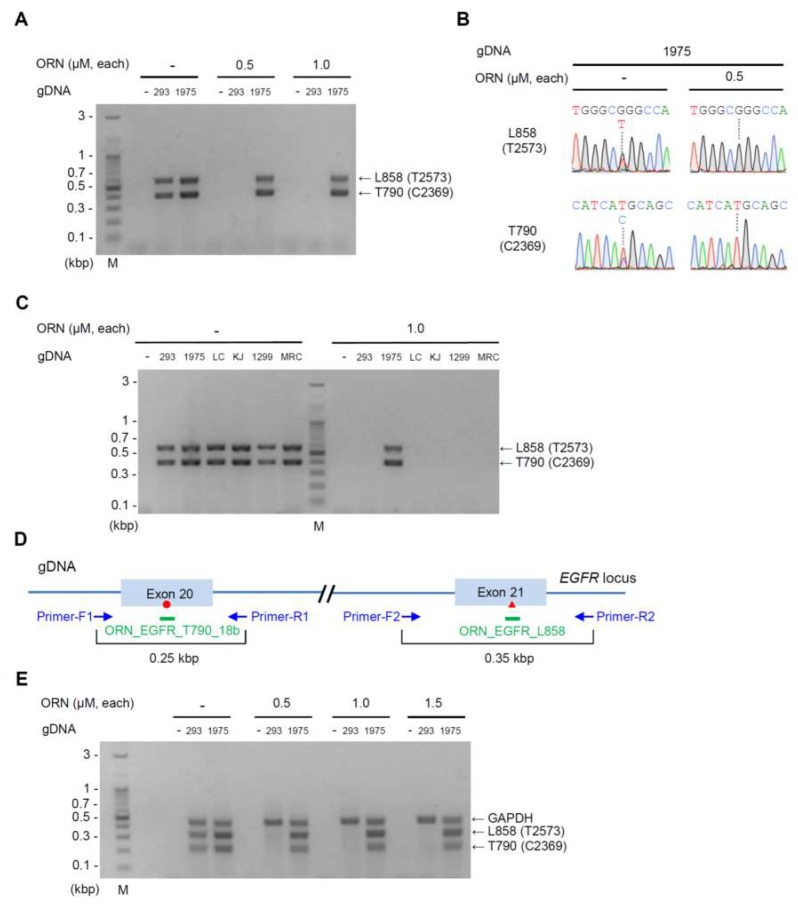
Multiplex ORNi-PCR for simultaneous detection of the T790M (C2369T) and L858R (T2573G) mutations in gDNA. (**A**) Results of multiplex ORNi-PCR with ORN_EGFR_T790_18b and ORN_EGFR_L858. Primer sets shown in Figure 2B and Supplementary Figure S1B were used simultaneously. (**B**) Results of DNA sequencing analysis. Amplicons of PCR or ORNi-PCR (0.5 µM each ORN) shown in (**A**) were subjected to DNA sequencing analysis. Sequencing signals around T790 (C2369) and L858 (T2573) are shown. (**C**) Results of multiplex ORNi-PCR with gDNA extracted from various lung cell lines. (**D**) Primer positions for multiplex ORNi-PCR combined with an internal control PCR. Primer positions for multiplex ORNi-PCR are shown. Primers for amplification of the *GAPDH* gene were used as an internal control PCR. The red circle and triangle represent the T790M (C2369T) and L858R (T2573G) mutations, respectively. (**E**) Results of multiplex ORNi-PCR combined with internal control PCR.

We next investigated whether ORNi-PCR could enable simultaneous detection of the T790M (C2369T) and L858R (T2573G) mutations in gDNA. The two mutation sites are 10 kbp apart from each other (Figure 1B) and it would be difficult to steadily amplify such a long sequence across both mutation sites using only one pair of primers. Therefore, we utilized multiplex ORNi-PCR to amplify two target sequences across each mutation site in one reaction tube. In this context, we used two primer sets to amplify 0.4 kbp across the T790M (C2369T) mutation (Figure 2B) and 0.6 kbp across the L858R (T2573G) mutation (Supplementary Figure S1B). We performed multiplex ORNi-PCR based on the optimal conditions for ORNi-PCR with ORN_EGFR_L858 and ORN_EGFR_T790_18b (Figure 2C). Multiplex PCR in the absence of ORNs amplified 0.4 and 0.6 kbp products from 293T and NCI-H1975 gDNA (Figure 3A). By contrast, in the presence of ORN_EGFR_L858 and ORN_EGFR_T790_18b, those amplifications were completely suppressed when using gDNA from 293T, but not gDNA from NCI-H1975. Thus, we confirmed that each ORN specifically suppressed DNA amplification across each target sequence (Supplementary Figure S2). DNA sequencing analysis of the PCR and ORNi-PCR amplicons shown in Figure 3A (0.5 µM each ORN) revealed that only the mutated sequences were detectable in the ORNi-PCR amplicons (Figure 3B).

We next evaluated the multiplex ORNi-PCR method with gDNA extracted from four other lung cell lines, RERF-LC-KJ, LC-2/ad, NCI-H1299, and MRC-5, of which only LC-2/ad harbors the *EGFR* L858R (T2573G) mutation [18,22,23,24]. However, multiplex ORNi-PCR amplified the 0.4 and 0.6 kbp products only from NCI-H1975 gDNA (Figure 3C). Although we sequenced the PCR products from LC-2/ad gDNA, we detected only the wild-type signals (Supplementary Figure S3), consistent with the results of multiplex ORNi-PCR (Figure 3C). These results suggest that ORNi-PCR can enable simultaneous detection of the T790M (C2369T) and L858R (T2573G) mutations in the *EGFR* gene.

To eliminate the possibility that the lack of amplification in ORNi-PCR was due to simple PCR failure, it would be useful to combine ORNi-PCR with an internal control PCR that amplifies an irrelevant DNA region. Amplification of the irrelevant DNA region would serve as a positive control for the ORNi-PCR. Accordingly, we designed a primer set to amplify a 0.5 kbp region of the *GAPDH* gene as an internal control PCR. In addition, we re-designed primer sets to amplify 0.25 kbp across the T790M (C2369T) mutation and 0.35 kbp across the L858R (T2573G) mutation (Figure 3D). As shown in Figure 3E, multiplex ORNi-PCR combined with an internal control PCR was feasible with ORN_EGFR_L858 and ORN_EGFR_T790_18b.

### 2.4. Simultaneous Detection of the T790M (C2369T) and L858R (T2573G) Mutations by ORNi-PCR with cDNA

**Figure 4:**
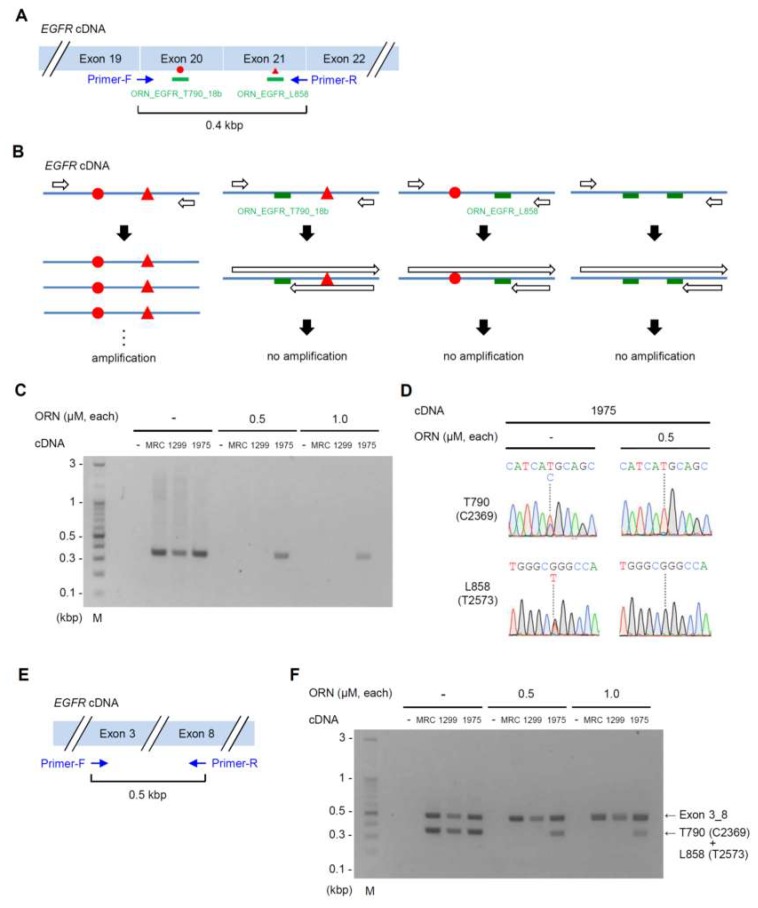
ORNi-PCR for simultaneous detection of the T790M (C2369T) and L858R (T2573G) mutations in cDNA. (**A**) Primer positions for ORNi-PCR on cDNA. ORN_EGFR_T790_18b and ORN_EGFR_L858 were used simultaneously. (**B**) Schematic diagram of ORNi-PCR with cDNA. If *EGFR* cDNA harbors the T790M (C2369T) and L858R (T2573G) mutations, the ORNs cannot hybridize with the cDNA, resulting in target amplification. By contrast, if cDNA does not possess either of the two mutations, an ORN would hybridize with the cDNA, resulting in suppression of target amplification. Red circle and triangle represent the T790M (C2369T) and L858R (T2573G) mutations, respectively. (**C**) Results of ORNi-PCR with cDNA. (**D**) Results of DNA sequencing analysis. Amplicons of PCR or ORNi-PCR (0.5 µM each ORN) shown in (**C**) were subjected to DNA sequencing analysis. Sequencing signals around T790 (C2369) and L858 (T2573) are shown. (**E**) Primer positions for an internal control PCR. (**F**) Results of ORNi-PCR combined with an internal control PCR.

We next applied the optimal experimental conditions for ORNi-PCR with gDNA to cDNA. For this purpose, we designed a primer set to amplify a 0.4 kbp region including the T790 (C2369) and L858 (T2573) positions on the *EGFR* cDNA (Figure 4A). Under this scheme, if neither ORN_EGFR_T790_18b nor ORN_EGFR_L858 is hybridized with its complementary sequences, the 0.4 kbp region would be amplified. By contrast, if one of the ORNs is hybridized, the 0.4 kbp region would not be amplified (Figure 4B). Therefore, ORNi-PCR with ORN_EGFR_T790_18b and ORN_EGFR_L858 can amplify the *EGFR* cDNA only if the T790M (C2369T) mutation is introduced into the same allele where the de novo L858R (T2573G) mutation was introduced. In this experiment, we subjected RNA extracted from MRC-5, NCI-H1299, and NCI-H1975 to reverse transcription (RT). Beforehand, we confirmed that the wild-type *EGFR* gene was transcribed in MRC-5 and NCI-H1299 cells (Supplementary Figure S4). PCR amplified the 0.4 kbp regions from cDNAs of all cell lines, whereas ORNi-PCR with ORN_EGFR_T790_18b and ORN_EGFR_L858 amplified the region only from NCI-H1975 cDNA (Figure 4C). To confirm the suppression events, we subjected PCR and ORNi-PCR amplicons from NCI-H1975 cDNA to DNA sequencing analysis (Figure 4D). DNA sequencing of the PCR amplicon yielded signals corresponding to both the wild-type and mutated *EGFR* sequences, suggesting that the mutated *EGFR* is transcribed in NCI-H1975. By contrast, DNA sequencing of the ORNi-PCR amplicon (0.5 μM each ORN) yielded signals corresponding only to the mutated *EGFR* sequence. Using this approach, we also confirmed that the T790M (C2369T) and L858R (T2573G) mutations are encoded by the same transcripts (Supplementary Figure S5), suggesting that both mutations exist on the same allele. We then designed a primer set for the internal control PCR, which amplified a 0.5 kbp region spanning exons 3 through 8 of the *EGFR* cDNA (Figure 4E). Thus, it was feasible to combine an internal control PCR with ORNi-PCR using ORN_EGFR_L858 and ORN_EGFR_T790_18b (Figure 4F).

In the case of simultaneous detection of the T790M (C2369T) and L858R (T2573G) mutations in the same *EGFR* cDNA by ORNi-PCR, it is important to demonstrate that the target sequence would not be amplified if one of the ORNs were hybridized to the corresponding cDNA sequence (Figure 4B). To this end, we designed ORN_EGFR_Ex20, an ORN complementary to the wild-type *EGFR* cDNA sequence between T790 (C2369) and L858 (T2573) (Supplementary Figure S6A,B). We sought to determine the practical T_m_ for ORN_EGFR_Ex20 by ORNi-PCR with NCI-H1975 gDNA. ORNi-PCR revealed that the practical T_m_ was also 59–62 °C (Supplementary Figure S6C), the same as those of ORN_EGFR_T790_18b and ORN_EGFR_L858. Next, when ORN_EGFR_Ex20 was used instead of ORN_EGFR_T790_18b or ORN_EGFR_L858, amplification of the *EGFR* sequence (0.4 kbp) was completely suppressed, even from NCI-H1975 cDNA (Supplementary Figure S7). These results suggest that, in the case of targeting multiple nucleotide mutations in one cDNA, ORNi-PCR can amplify the target DNA sequence only when neither ORN is hybridized to its target sequences. In other words, amplification by ORNi-PCR indicates that multiple target nucleotide mutations are present on one cDNA. Together, these results demonstrated that ORNi-PCR with cDNA can simultaneously detect the T790M (C2369T) and L858R (T2573G) mutations in the *EGFR* gene in the same allele.

### 2.5. Sensitivity of ORNi-PCR for Simultaneous Detection of Two EGFR Mutations

**Figure 5:**
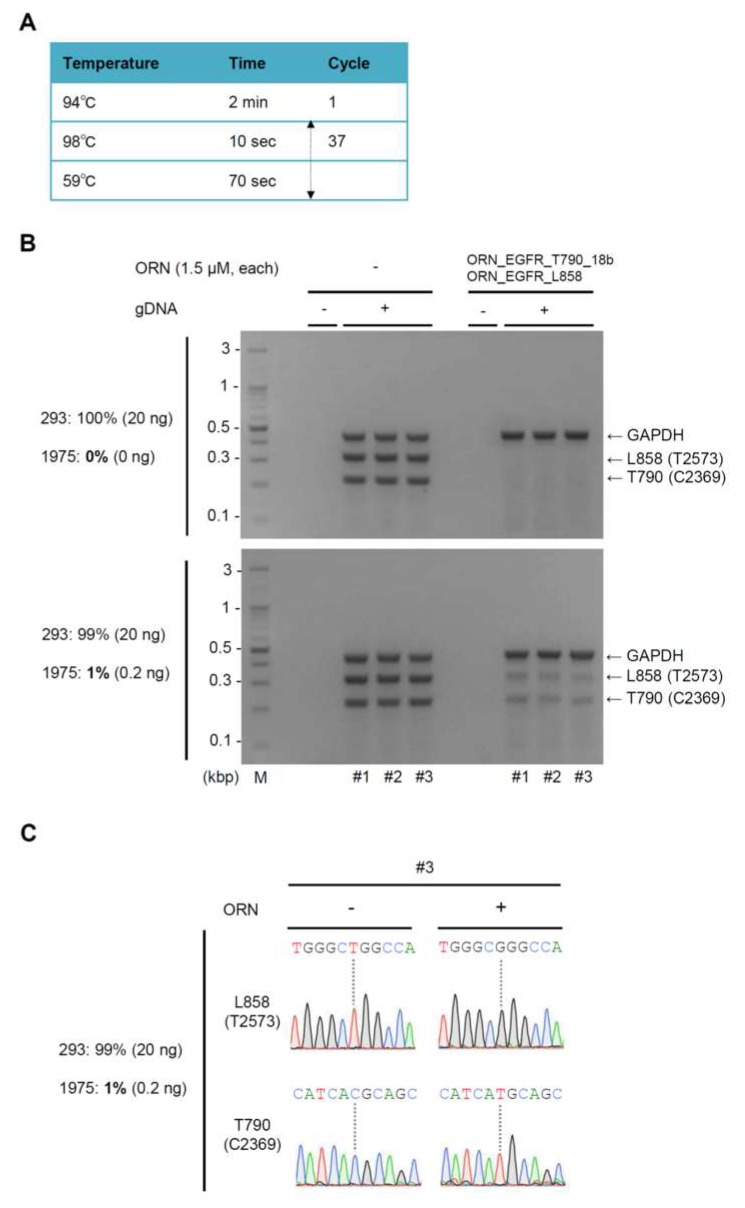
Sensitivity of multiplex ORNi-PCR for simultaneous detection of the T790M (C2369T) and L858R (T2573G) mutations in gDNA. (**A**) Conditions for multiplex ORNi-PCR with gDNA. (**B**) Results of ORNi-PCR with gDNA. Based on Figure 3E, 1.5 μM each of ORN_EGFR_T790_18b and ORN_EGFR_L858 were used simultaneously. Either 293T gDNA (upper panel) or 293T gDNA mixed with NCI-H1975 gDNA (lower panel) was used for multiplex ORNi-PCR combined with an internal control PCR. Results of triplicate experiments are shown. (**C**) Results of DNA sequencing analysis. PCR or ORNi-PCR amplicons shown in (**B**, lower panel) were subjected to DNA sequencing analysis. Sequencing signals from PCR (#3) and ORNi-PCR (#3) around T790 (C2369) and L858 (T2573) are shown.

Next, we sought to determine the sensitivity with which ORNi-PCR can detect multiple single-nucleotide mutations. To this end, we mixed 293T gDNA with NCI-H1975 gDNA so that NCI-H1975 gDNA constituted 0.2% or 1% of the total. In order to be able to clearly detect such a small population of mutated *EGFR* by electrophoresis, we performed ORNi-PCR (including an internal control PCR) with 37 cycles of denaturing and annealing/elongation steps (Figure 5A). Multiplex ORNi-PCR with ORN_EGFR_T790_18b and ORN_EGFR_L858 completely suppressed amplification of the wild-type *EGFR* sequences (0.35 and 0.25 kbp) from 293T gDNA (Figure 5B). By contrast, when NCI-H1975 gDNA constituted 1% of total gDNA, 0.35 kb and 0.25 kbp amplicons were observed even in the presence of the ORNs (Figure 5B). However, no such amplification was observed when NCI-H1975 gDNA constituted only 0.2% of total gDNA (Supplementary Figure S8). We added three more cycles of denaturing and annealing/elongation steps, but specific amplification was still not observed. DNA sequencing analysis of the PCR and ORNi-PCR amplicons from the gel shown in Figure 5B revealed only the mutated *EGFR* sequences from the ORNi-PCR amplicons (Figure 5C and Supplementary Figure S9). These results demonstrated that multiplex ORNi-PCR with gDNA can simultaneously detect the T790M (C2369T) and L858R (T2573G) mutations so long as the gDNA harboring these mutations constitutes 1% or more of the total gDNA in the sample.

**Figure 6:**
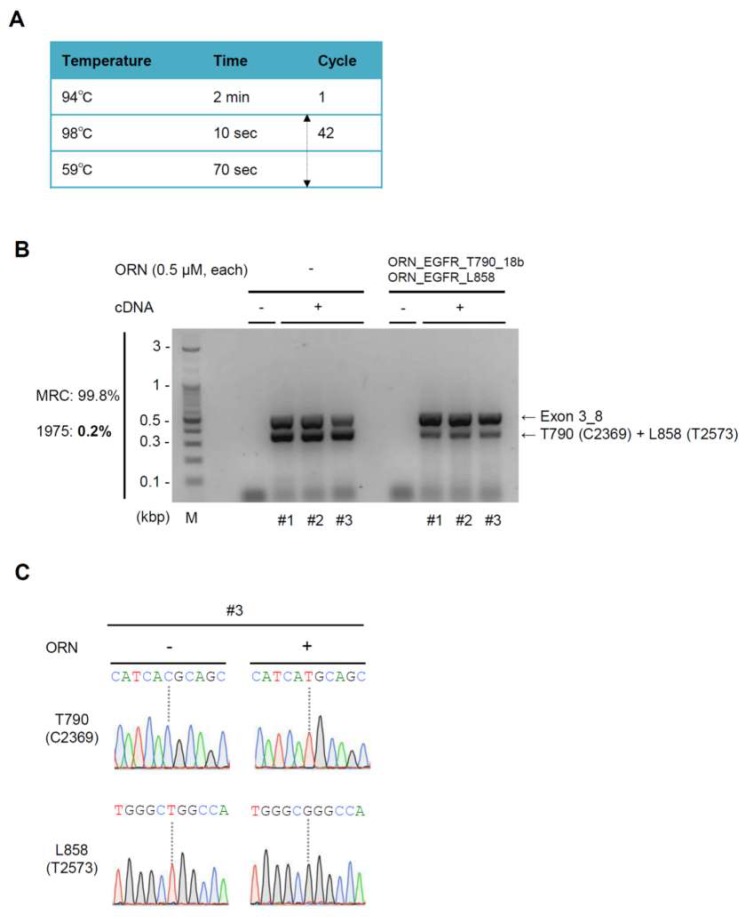
Sensitivity of ORNi-PCR for simultaneous detection of the T790M (C2369T) and L858R (T2573G) mutations in cDNA. (**A**) Conditions for ORNi-PCR with cDNA. (**B**) Results of ORNi-PCR with cDNA. Based on Figure 4F, 0.5 µM each of ORN_EGFR_T790_18b and ORN_EGFR_L858 were used simultaneously. cDNA reverse-transcribed from RNA extracted from a mixture of MRC-5 cells and NCI-H1975 cells was used for ORNi-PCR in combination with an internal control PCR. Results of triplicate experiments are shown. (**C**) Results of DNA sequencing analysis. PCR or ORNi-PCR amplicons present in (**B**) were subjected to DNA sequencing analysis. Sequencing signals from PCR (#3) and ORNi-PCR (#3) around T790 (C2369) and L858 (T2573) are shown.

We also examined the sensitivity of ORNi-PCR on cDNA. In this experiment, we mixed MRC-5 cells and NCI-H1975 cells, such that NCI-H1975 cells constituted 0.2% or 1% of the total cell number, and then extracted total RNA. After the RT reactions, cDNA was subjected to ORNi-PCR with 0.5 μM each of ORN_EGFR_T790_18b and ORN_EGFR_L858 (Supplementary Figure S10A). ORNi-PCR completely suppressed amplification of the wild-type *EGFR* sequences (0.4 kbp) from MRC-5 cDNA (upper panel in Supplementary Figure S10B). By contrast, when NCI-H1975 constituted 1% of the total cell number, the 0.4 kbp sequence was amplified even in the presence of the ORNs (lower panel in Supplementary Figure S10B). DNA sequencing analysis detected only the mutated *EGFR* sequences from the ORNi-PCR amplicons (Supplementary Figure S10C). We observed similar results when NCI-H1975 constituted 0.2% of the total cell number (Supplementary Figure S11A). Under these conditions, DNA sequencing data yielded modest noise signals, probably due to the low concentration of the target DNA (Supplementary Figure S11B). When we added five more cycles of denaturing and annealing/elongation steps, ORNi-PCR amplified higher levels of the target products, and noise signals were not detected in DNA sequencing analysis (Figure 6). Thus, ORNi-PCR with cDNA can simultaneously detect the two *EGFR* single-nucleotide mutations if cancer cells harboring these mutations constitute more than 0.2% of the total cell number.

Taken together, these findings demonstrate that ORNi-PCR can simultaneously detect two single-nucleotide mutations, T790M (C2369T) and L858R (T2573G), in the same allele of the *EGFR* gene. Moreover, we learned that cDNA is more suitable for detection of these mutations, and yields higher sensitivity than gDNA.

## 3. Discussion

In this study, we showed that ORNi-PCR is an accurate method for simultaneously detecting multiple single-nucleotide mutations in the *EGFR* gene. Various PCR-based methods have been developed to detect mutations of interest. For example, blocking PCR using LNAs or PNAs has been used to detect *EGFR* mutations for clinical diagnosis [25,26,27]. In bronchoscopic specimens, this approach can individually distinguish more than ten types of mutations in the *EGFR* gene [28,29]. However, LNAs and PNAs are costly and some limits on the design of PNAs have been reported [30]. By contrast, no such limitations apply to ORNs. Thus, ORNi-PCR should be especially attractive when suitable PNAs (and/or LNAs) cannot be designed. In addition, the cost of ORNi-PCR is less than one tenth of that of PNA-LNA clamp PCR. On the other hand, DNA can also be used as a blocker in blocking PCR [16,17,31]. In this context, the 3′-end of blocker DNA is modified (e.g., by dideoxygenation or phosphorylation) to block extension from the blocker DNA. Alternatively, 3′-nucleotide(s) can be designed to be non-complementary with the target templates. In this regard, however, some high-fidelity DNA polymerases have 3′→5′ exonuclease activity and remove 3′-modifications of blocker DNA. To avoid removal of such 3′-modifications, DNA polymerases lacking 3′→5′ exonuclease activity can be used, although this sacrifices high-fidelity PCR amplification. In addition, such 3′-end modifications cannot completely block extension from the blocker DNA [32].

ORNs have a significant advantage in the blocking event. It is not necessary to modify their 3′-ends because they cannot serve as primers under PCR conditions [33]. In addition, DNA–RNA duplexes are more stable than DNA–DNA double strands. As ORNi-PCR uses α-type DNA polymerase possessing 3′→5′ exonuclease activity (i.e., proof-reading activity), ORNi-PCR can more precisely amplify target DNA. Our results showed that ORNi-PCR can detect lung cancer cells harboring two single-nucleotide *EGFR* mutations among a large number of cells harboring the wild-type *EGFR* sequence, even when the cancer cells constitute only 0.2% of the total cell number (Figure 6 and Supplementary Figures S11 and S12). Consequently, the sensitivity of ORNi-PCR is comparable to that of PNA–LNA PCR clamp methods [18,34]. We previously described a simple rule for the design of ORNs with their T_m_s [21]. To establish multiplex ORNi-PCR for the discrimination of multiple single-nucleotide mutations, it is important to adjust the practical T_m_s of ORNs. We showed that the practical T_m_s of ORNs can easily be adjusted by following this simple rule (Figure 2 and Supplementary Figure S6).

In this study, we included an internal positive control PCR to confirm the success of the ORNi-PCR. In clinical diagnoses, gDNA or RNA extracted from fixed cells, such as formalin fixed paraffin-embedded (FFPE) specimens, biopsy specimens, or blood samples, is used as a template. However, such templates may be damaged and therefore unsuitable for PCR or RT-PCR, even if only a few hundred base pairs are to be amplified [35]. In such cases, ORNi-PCR combined with an internal control PCR would represent a more reliable and advantageous method.

We used various cell lines, including lung cancer cells in this study. We did not observe the L858R (T2573G) mutation in LC-2/ad (Figure 3), although PCR clamping has demonstrated the existence of this mutation in both LC-2/ad and RERF-LC-A1 [18]. Notably in this regard, other studies also reported no L858R (T2573G) mutation in LC-2/ad and RERF-LC-A1 [36,37]. Consequently, we could only use NCI-H1975 as a cell line harboring the L858R (T2573G) mutation in addition to T790M (C2369T). Using ORN_EGFR_Ex20, we proved that ORNi-PCR with cDNA detects positive signals only when both the T790M (C2369T) and L858R (T2573G) mutations are introduced in the same allele (Supplementary Figure S7). The T790M (C2369T) mutation mainly arises in conjunction with acquired resistance against the first-generation EGFR-TKI inhibitors used against L858R-mutated or exon 19-deleted EGFR. Therefore, ORNi-PCR with cDNA would be especially useful for monitoring the appearance of the T790M (C2369T) mutation during treatment with first-generation EGFR-TKIs. By contrast, if T790M (C2369T) and L858R (T2573G) are introduced as de novo mutations in the *trans* position, ORNi-PCR using ORNs targeting T790 (C2369) and L858 (T2573) sites will not amplify the *EGFR* sequence from cDNA, as expected from Supplementary Figure S7. However, even in such a case, ORNi-PCR with gDNA can detect both mutations because each mutation is evaluated independently using each primer set (Figure 3). Alternatively, it is possible to use each ORN for ORNi-PCR with cDNA to individually detect T790M (C2369T) and L858R (T2573G) in *EGFR* cDNA. Thus, by combining gDNA, cDNA, ORNs, and primer sets, it is possible to flexibly evaluate whether T790M (C2369T) and L858R (T2573G) mutations were introduced in *cis* or in *trans*. As for Ex19 Del, 9–20 nucleotides are generally deleted in exon 19 and a few nucleotides are additionally substituted or inserted in some cases. As (1) ORNi-PCR can clearly discriminate single-nucleotide mutations and (2) 9–20 nucleotide mismatches occur between Ex19 Del and an ORN targeting the wild-type exon 19 sequence, we think that detection of Ex19 Del is much easier than that of T790M or L858R. In addition, based on the present study, other *EGFR* minor mutations (e.g., G719X, L861Q, S768I) can also be simultaneously detected. On the other hand, ORNi-PCR-based methods may also be applicable to the detection of multiple single-nucleotide mutations in other genes. For example, the use of cetuximab, an anti-*EGFR* antibody, for treatment of colorectal cancers is limited to patients who do not harbor genetic mutations in the *RAS* genes [10,38]. Multiple major and minor *RAS* mutations have been identified, and analysis of such *RAS* mutations is essential before prescription of cetuximab. Multiplex ORNi-PCR could be used to detect *RAS* mutations. In the future, it will be interesting to apply ORNi-PCR to mutations in other genes, as well as clinical diagnosis of *EGFR* mutations.

NGS analysis can identify unknown nucleotide mutations in cancer cells. However, in clinical diagnosis, it may be more valuable to sensitively identify defined driver mutations because targeted medicines are usually prepared with such mutations in mind. In addition, some major driver mutations are mutually exclusive and decisive for treatment policy. In this regard, targeted NGS panels may be more useful than whole genome sequencing. If ORNs can be designed for targeted NGS panels, defined mutations may be more sensitively and effectively detected without amplification of wild-type sequences. Alternatively, it might be possible to use ORNi-PCR as a first screening tool for detection of major driver mutations. In the case of no detectable major mutations, NGS analysis could be employed as a second screening tool. Such a step-by-step approach might reduce the total cost of clinical diagnosis. Thus, combinatorial use of ORNi-PCR and NGS analysis may also be attractive and advantageous.

## 4. Materials and Methods

### 4.1. Oligonucleotides

ORNs were chemically synthesized (FASMAC, Tokyo, Japan). ORNs and primers are listed in Supplementary Table S1.

### 4.2. Cell Culture

NCI-H1975, NCI-H1299, and MRC-5 were purchased from American Type Culture Collection (Manassas, VA, USA). LC-2/ad and RERF-LC-KJ were provided by the RIKEN BioResource Center (Tsukuba, Japan) through the National Bio-Resource Project of the Ministry of Education, Science, Sports and Culture of Japan. NCI-H1975, NCI-H1299, and RERF-LC-KJ were cultured in RPMI-1640 (Wako, Osaka, Japan) supplemented with 10% fetal bovine serum (FBS). MRC-5 was cultured in Modified Eagle Medium (Wako, Osaka, Japan) supplemented with 10% FBS. LC-2/ad was cultured in a 1:1 mixture of HamF12 (Wako, Osaka, Japan) and RPMI-1640 supplemented with 25 mM HEPES and 15% FBS.

### 4.3. ORNi-PCR with gDNA

gDNA was extracted by a standard phenol/chloroform protocol. After measurement of its concentration, the gDNA was diluted and used in each experiment. ORNi-PCR was performed as described previously [21]. Briefly, KOD-Plus-Ver. 2 (Toyobo, Osaka, Japan), 20 pg–20 ng of gDNA, 0.3 μM of each primer, and 0.5–2 μM ORNs were mixed in a total volume of 10 μL. Reactions conditions were as follows: A temperature of 94 °C for 2 min, followed by 30–42 cycles at 98 °C for 10 s and 56–62 °C for 70–80 s.

### 4.4. Electrophoresis and DNA Sequencing Analysis

PCR or ORNi-PCR products were electrophoresed on 2% or 3% agarose gels. If necessary, the products were purified from the gels and subjected to DNA sequencing analysis (Eurofins Genomics, Tokyo, Japan). DNA sequencing data were analyzed using SnapGene Viewer (https://www.snapgene.com/snapgene-viewer/).

### 4.5. RNA Extraction, RT Reactions, and Two-Step ORNi-PCR with cDNA

Total RNA was extracted with RNeasy Mini Kit (Qiagen, Hilden, Germany). RNA (25 ng) was used for RT using ReverTra Ace qPCR RT Master Mix (Toyobo, Osaka, Japan) in a 10 μL reaction volume. After the RT reaction, ORNi-PCR was performed with 1 μL of cDNA as described above for gDNA.

## 5. Patents

T.F. and H.F. have filed patent applications related to ORNi-PCR, as follows: (1) Name: Method for suppressing amplification of specific nucleic acid sequences; Patent applicant: Osaka University; Name of inventors: H.F., T.F., Naoki Tanigawa; Number: Japanese Patent Application No. 2014-176018 and 2015-165643; Status: Under review; Specific aspect of manuscript covered in patent application: The patent application covers the basic principle of the ORNi-PCR used in the manuscript. (2) Name: Method for detecting differences in target nucleic acid region; Patent applicant: Hirosaki University; Name of inventors: H.F., T.F.; Number: Japanese Patent Application No. 2018-81752 and PCT/JP2019/016843; Status: filed; Specific aspect of manuscript covered in patent application: The patent application covers application of ORNi-PCR to detection of nucleotide mutations using the two-step protocol.

## Supplementary Materials

Supplementary materials can be found at https://www.mdpi.com/1422-0067/20/16/4020/s1.

## Abbreviations

EGFR	Epidermal Growth Factor Receptor
gDNA	genomic DNA
cDNA	complementary DNA
PCR	Polymerase Chain Reaction
ORNi-PCR	Oligoribonucleotide interference-PCR
