# Supplementary material for: Simultaneous Detection of the T790M and L858R Mutations in the EGFR Gene by Oligoribonucleotide Interference-PCR
Source: Int J Mol Sci. 2019 Aug 17;20(16):4020. doi: 10.3390/ijms20164020 (PMC6720885; doi:10.3390/ijms20164020)
Supplement: Supplementary file 1 [file ijms-20-04020-s001.pdf]

Table S1. ORNs and primers

Types	Names	Sequences (5' to 3')	Experiments
ORNs	ORN_EGFR_T790_18b	ggcaugagcugcgugaug	Figs. 2D, 3A, 3C, 3E, 4F, 5B, and 6B, and Supplementary Figs. S2, S7D, S8, S10, and S11
	ORN_EGFR_T790_19b	gcaugagcugcgugaugag	Fig. 2D
	ORN_EGFR_T790_20b	ggcaugagcugcgugaugag	Fig. 2D
	ORN_EGFR_L858	caguuuggccagcccaaaauc	Figs. 3A, 3C, 3E, 4F, 5B, and 6B, and Supplementary Figs. S1D, S2, S7B, S8, S10, and S11
	ORN_EGFR_Ex20	caguugagcagguacuggg	Supplementary Figs. S6C, S7B, and S7D
Primers	hEGFR-T790M_L858R_cDNA-F3	ATGGCCAGCGTGGACAAC	Figs. 4C, 4F, and 6B, and Supplementary Figs. S10 and S11
	hEGFR-T790M_L858R_cDNA-R	TGATTCCAATGCCATCCACTTGA	Figs. 4C, 4F, and 6B, and Supplementary Figs. S10 and S11
	hEGFR-Exon3_8-cDNA-F	GCCCATGAGAAATTTACAGGAA	Figs. 4F and 6B, and Supplementary Figs. S10 and S11
	hEGFR-Exon3_8-cDNA-R	ACCACATAATTACGGGGACACTT	Figs. 4F and 6B, and Supplementary Figs. S10 and S11
	hEGFR-Exon20-F3	CTTCACAGCCCTGCGTAAACGTC	Figs. 2D, 3A, and 3C
	hEGFR-Exon20-R3	GCTCCTTATCTCCCCTCCCCGTA	Figs. 2D, 3A, and 3C
	hEGFR-Exon20-F4	CACACTGACGTGCCTCTCC	Figs. 3E and 5B, and Supplementary Figs. S2, S6C, and S8
	hEGFR-Exon20-R4	TCTCCCTTCCCTGATTACCTTT	Figs. 3E and 5B, and Supplementary Figs. S2, S6C, and S8
	hEGFR-Exon21-F2	AATTCGGATGCAGAGCTTCTT	Figs. 3E and 5B, and Supplementary Figs. S2 and S8
	hEGFR-Exon21-R2	CACCCAGAATGTCTGGAGAGC	Figs. 3E and 5B, and Supplementary Figs. S2 and S8
	hEGFR-Exon21-F	GCCTTTCCATTCTTTGGATCAG	Figs. 3A and 3C, and Supplementary Fig. S1D
	hEGFR-Exon21-R	CTGCAGGGAGAGACTGAAACCT	Figs. 3A and 3C, and Supplementary Fig. S1D
	hGAPDH-0.5kbp-F2	GCCTAGGGCTGCTCACATATTCT	Figs. 3E and 5B, and Supplementary Fig. S8
	hGAPDH-0.5kbp-R2	ACAGGACCATATTGAGGGACACA	Figs. 3E and 5B, and Supplementary Fig. S8

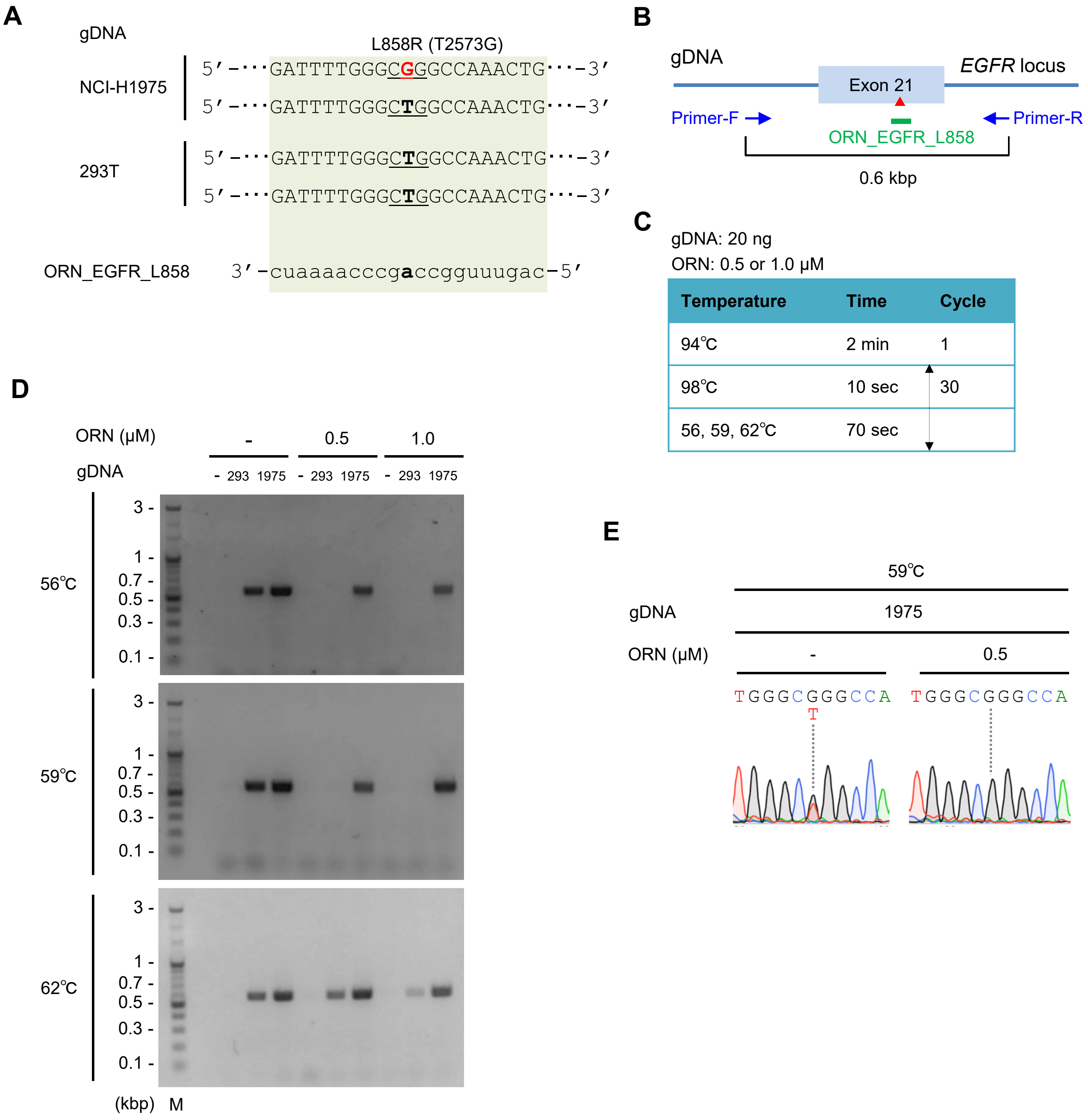

Figure S1. Detection of the L858R (T2573G) mutation by ORNi-PCR. (A) ORN_EGFR_L858 for ORNi-PCR. (B) Primer positions for ORNi-PCR. A red triangle represents the L858R (T2573G) mutation. (C) Conditions for two-step ORNi-PCR. (D) Results of ORNi-PCR. (E) Results of DNA sequencing analysis. PCR or ORNi-PCR amplicons present in (D) were subjected to DNA sequencing analysis. Sequencing signals around L858 (T2573) are shown.

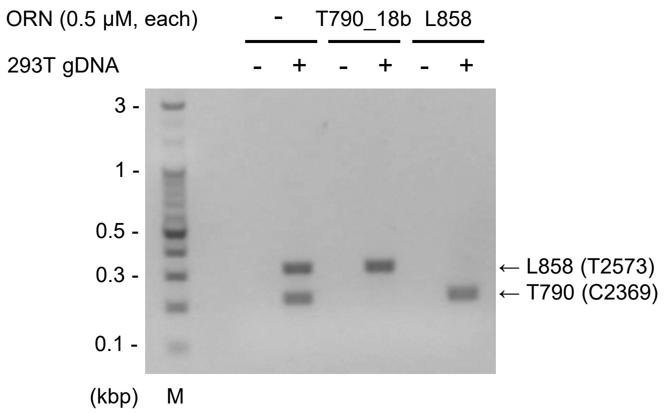

Figure S2. Sequence-specific suppression by ORNi-PCR. Two-step ORNi-PCR was performed in the presence of each ORN_EGFR_T790_18b or ORN_EGFR_L858. In this study, the primer sets used in Figure 3E were utilized.

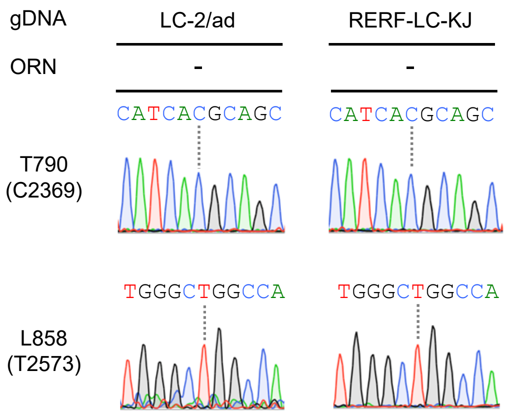

Figure S3. Results of DNA sequencing analysis. PCR amplicons present in Figure 3C (LC-2/ad and RERF-LC-KJ) were subjected to DNA sequencing analysis. Sequencing signals around T790 (C2369) and L858 (T2573) are shown.

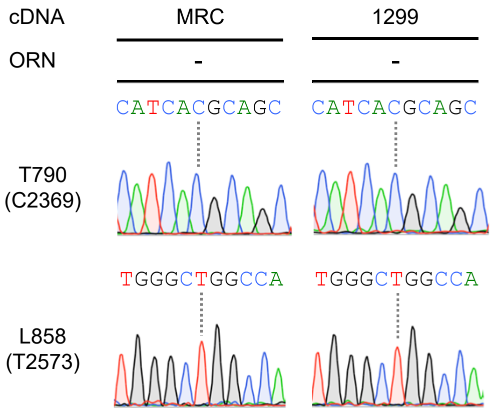

Figure S4. Results of DNA sequencing analysis. PCR amplicons present in Figure 4C (MRC-5 and NCI-H1299) were subjected to DNA sequencing analysis. Sequencing signals around T790 (C2369) and L858 (T2573) are shown.

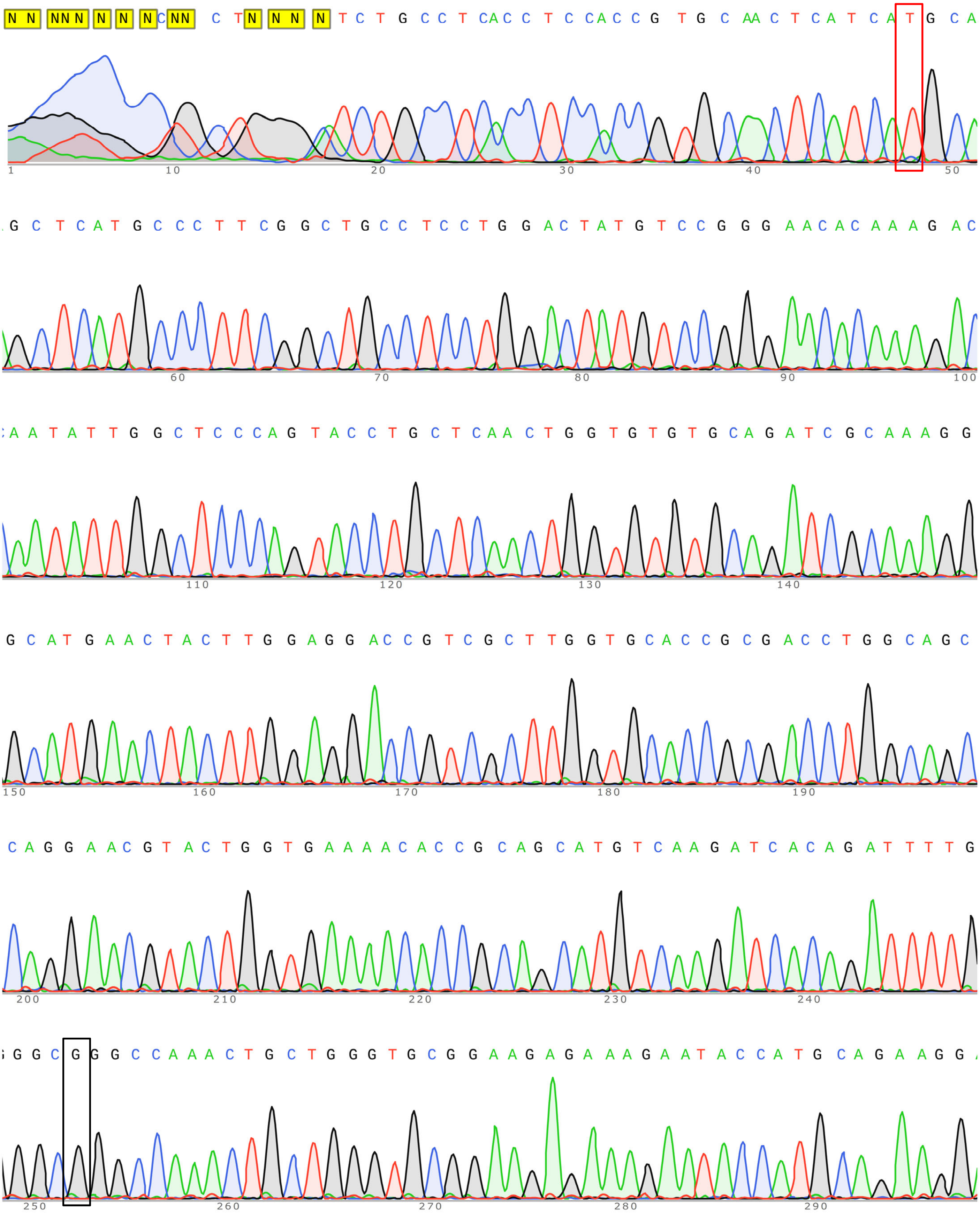

Figure S5. The full image of DNA sequencing signals of the ORNi-PCR amplicon shown in Figure 4D. After extraction of the DNA sequencing data as a PDF, the full image of DNA sequencing signals was shown here. The sequencing signals corresponding to C2369T and T2573G are shown in red and black squares, respectively.

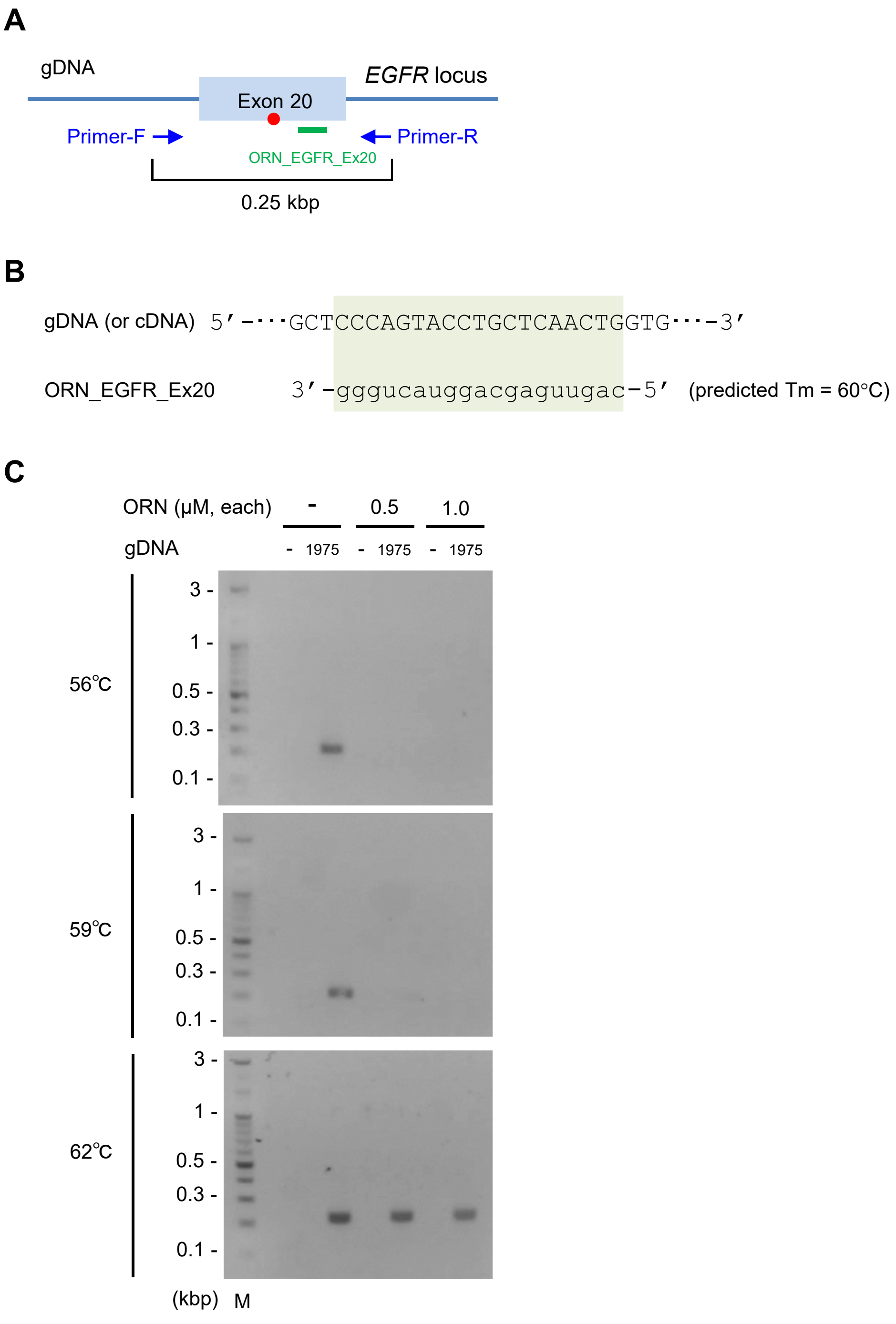

Figure S6. Evaluation of ORN_EGFR_Ex20. (A and B) ORN_EGFR_Ex20 designed to suppress amplification of the wild-type *EGFR* sequence in ORNi-PCR. A red circle represents the T790M (C2369T) mutation. (C) Results of ORNi-PCR. Two-step ORNi-PCR was performed as shown in Figure 2C.

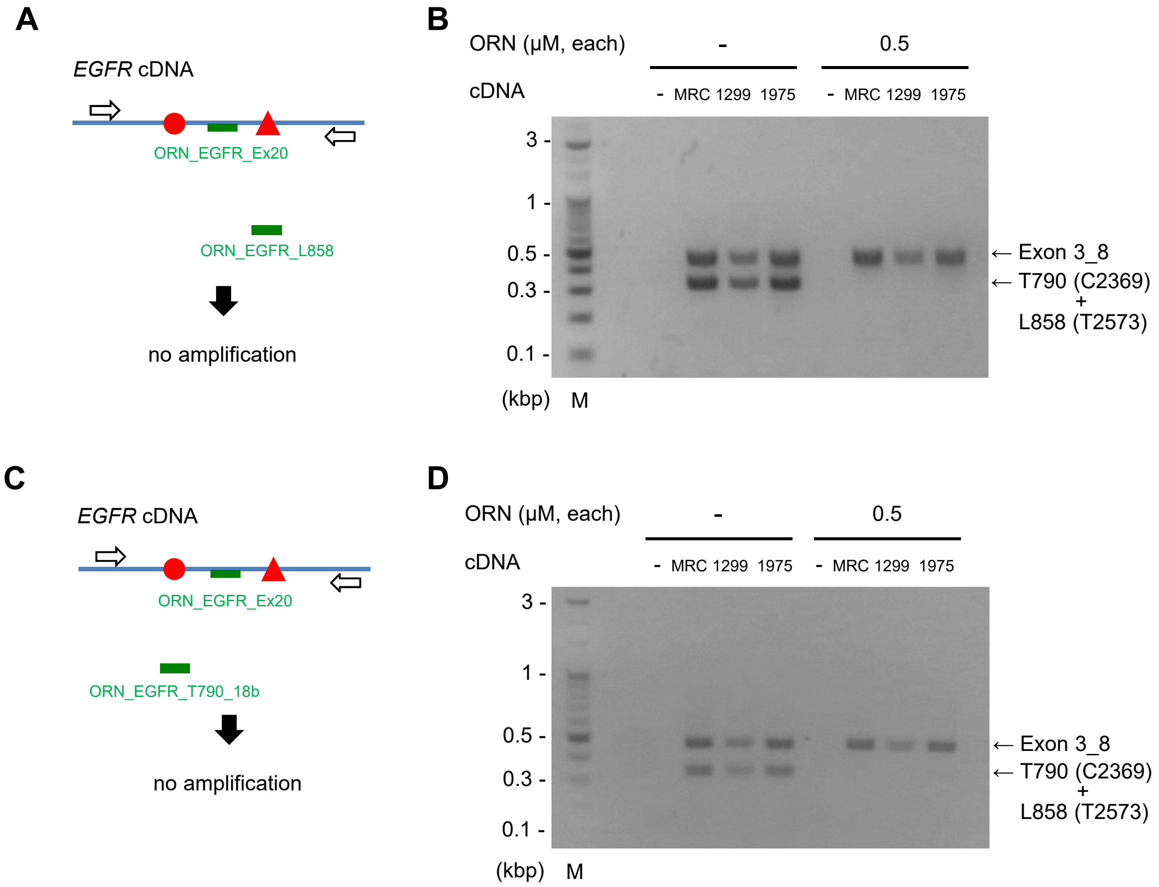

Figure S7. ORNi-PCR with ORN_EGFR_Ex20. (A) Schematic diagram of ORNi-PCR with ORN_EGFR_Ex20 and ORN_EGFR_L858. (B) Results of ORNi-PCR with ORN_EGFR_Ex20 and ORN_EGFR_L858. (C) Schematic diagram of ORNi-PCR with ORN_EGFR_Ex20 and ORN_EGFR_T790_18b. (D) Results of ORNi-PCR with ORN_EGFR_Ex20 and ORN_EGFR_T790_18b. (A and C) A red circle and triangle represent the T790M (C2369T) and L858R (T2573G) mutations, respectively. (B and D) Two-step ORNi-PCR was performed as shown in Figure 2C. cDNA was used as templates. An internal control PCR was combined with ORNi-PCR.

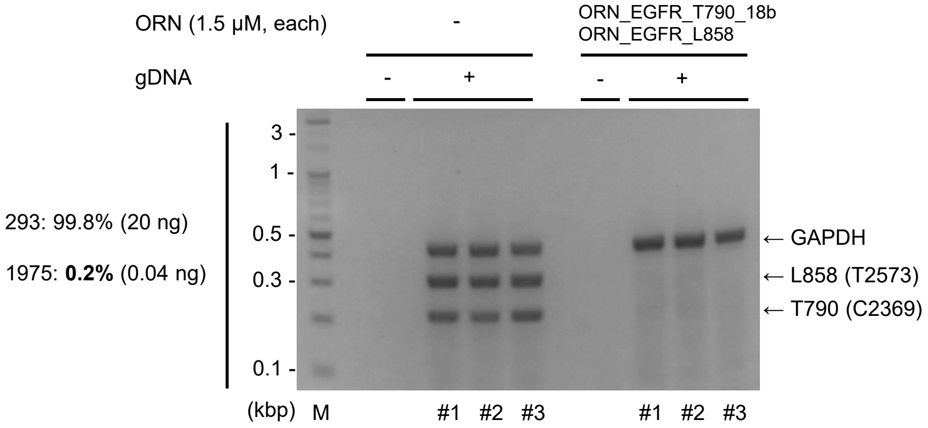

Figure S8. Sensitivity of ORNi-PCR for simultaneous detection of the T790M (C2369T) and L858R (T2573G) mutations in gDNA. Results of ORNi-PCR with gDNA. 293T gDNA mixed with NCI-H1975 gDNA was used for multiplex ORNi-PCR combined with an internal control PCR. Results of triplicate experiments are shown.

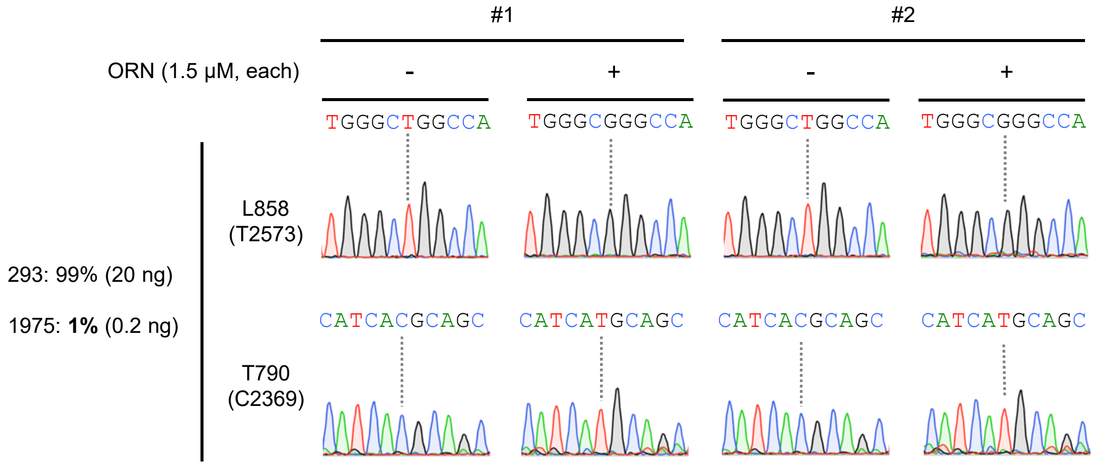

Figure S9. Results of DNA sequencing analysis. PCR or ORNi-PCR amplicons present in Figure 5B (Lower panel) were subjected to DNA sequencing analysis. Sequencing signals from PCR (#1 and #2) or ORNi-PCR (#1 and #2) are shown. Sequencing signals around T790 (C2369) and L858 (T2573) are shown.

A

Temperature	Time	Cycle
94℃	2 min	1
98℃	10 sec	37
59℃	70 sec	

B

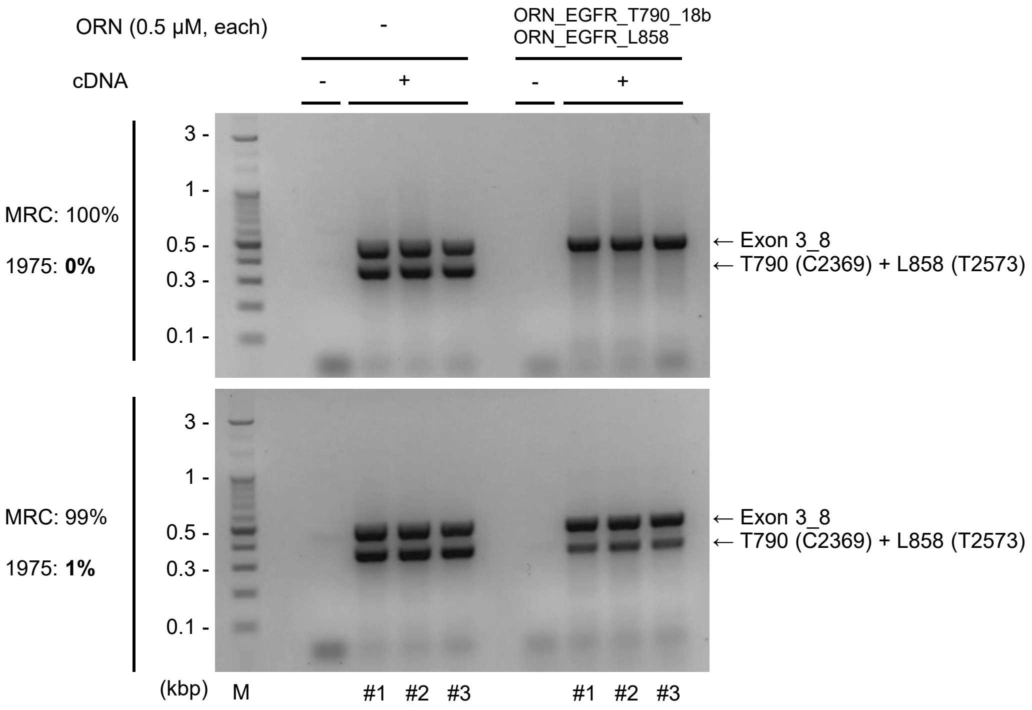

C

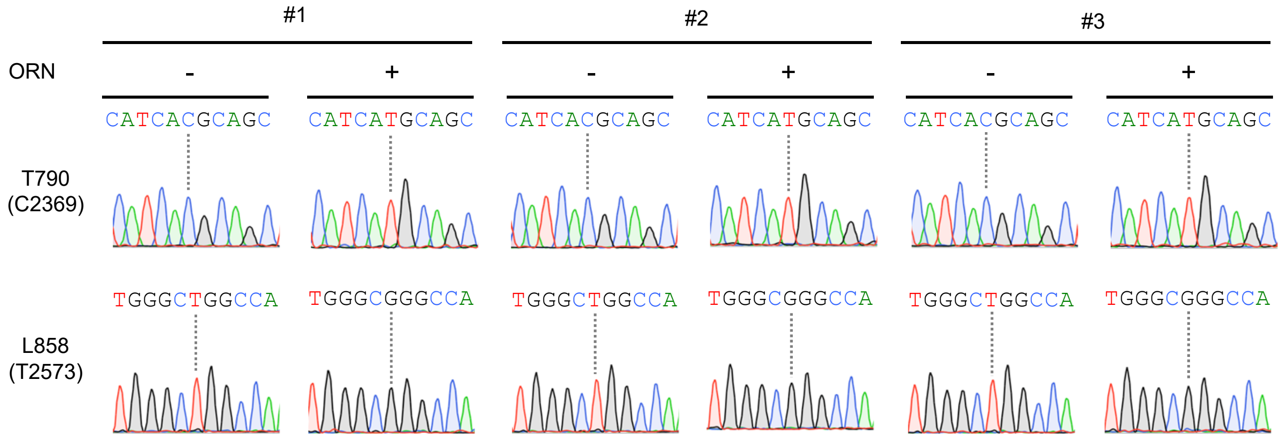

Figure S10. Sensitivity of ORNi-PCR for simultaneous detection of the T790M (C2369T) and L858R (T2573G) mutations in cDNA. (A) Conditions for ORNi-PCR with cDNA. (B) Results of ORNi-PCR with cDNA. Based on Figure 4F, 0.5 μM each of ORN_EGFR_T790_18b and ORN_EGFR_L858 were used simultaneously. cDNA reverse-transcribed from RNA extracted from MRC-5 cells (upper panel) or MRC-5 cells mixed with NCI-H1975 cells (lower panel) was subjected to ORNi-PCR combined with an internal control PCR. Results of triplicate experiments are shown. (C) Results of DNA sequencing analysis. PCR or ORNi-PCR amplicons present in (B, lower panel) were subjected to DNA sequencing analysis. Sequencing signals from PCR or ORNi-PCR around T790 (C2369) and L858 (T2573) are shown.

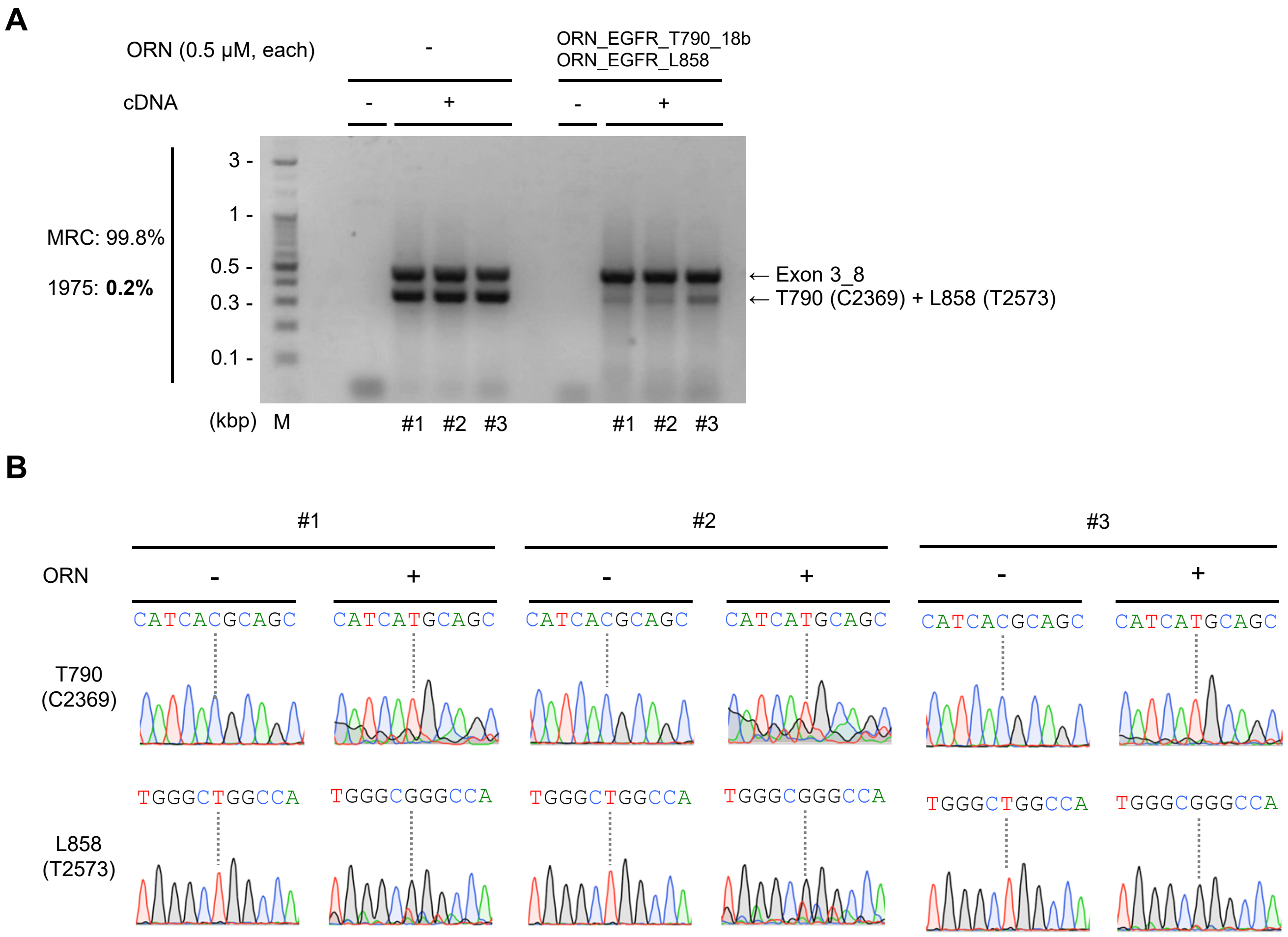

Figure S11. Sensitivity of ORNi-PCR for simultaneous detection of the T790M (C2369T) and L858R (T2573G) mutations in cDNA. (A) Results of ORNi-PCR with cDNA. cDNA reverse-transcribed from RNA extracted from MRC-5 cells mixed with NCI-H1975 cells was subjected to ORNi-PCR combined with an internal control PCR. Results of triplicate experiments are shown. (B) Results of DNA sequencing analysis. PCR or ORNi-PCR amplicons present in (A) were subjected to DNA sequencing analysis. Sequencing signals from PCR or ORNi-PCR around T790 (C2369) and L858 (T2573) are shown.

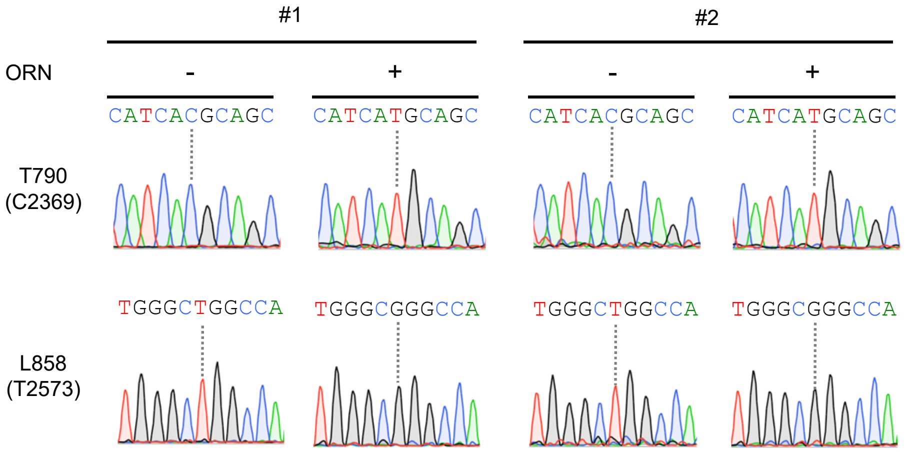

Figure S12. Results of DNA sequencing analysis. PCR or ORNi-PCR amplicons present in Figure 6B were subjected to DNA sequencing analysis. Sequencing signals from PCR (#1 and #2) or ORNi-PCR (#1 and #2) are shown. Sequencing signals around T790 (C2369) and L858 (T2573) are shown.
